# Delayed presentation of severe rhabdomyolysis leading to acute kidney injury following atorvastatin-gemfibrozil combination therapy: a case report

**DOI:** 10.1186/s13256-018-1685-0

**Published:** 2018-05-22

**Authors:** Chamara Dalugama, Manoji Pathirage, S. A. M. Kularatne

**Affiliations:** Department of Medicine, University of Peradeniya, Peradeniya, Sri Lanka

**Keywords:** Rhabdomyolysis, Acute kidney injury, Atorvastatin, Gemfibrozil, Hemodialysis, Myoglobin, Creatine kinase

## Abstract

**Background:**

Rhabdomyolysis is a rare but serious complication of lipid-lowering therapy. Statin and fibrate combination increases the risk of rhabdomyolysis possibly by pharmacodynamic interactions. Advanced age, diabetes, hypothyroidism, polypharmacy, and renal impairment are known to increase the risk of rhabdomyolysis. Management strategies include fluid resuscitation and urine alkalinization. Renal indications such as refractory hyperkalemia, acidosis, fluid overload, or uremic complications mandate renal replacement therapy in rhabdomyolysis.

**Case presentation:**

We report the case of a 62-year-old Sri Lankan Sinhalese man with dyslipidemia, type 2 diabetes mellitus with renal impairment, and hypothyroidism who was on atorvastatin; he was started on gemfibrozil and developed muscle symptoms. Although gemfibrozil was discontinued soon after, he presented with rhabdomyolysis with acute kidney injury 1 month later. He needed hemodialysis due to refractory hyperkalemia, metabolic acidosis, and fluid overload.

**Conclusions:**

Rhabdomyolysis is a rare but serious complication due to lipid-lowering therapy with statins and fibrates. Treating physicians should be aware and patients should be warned to report about muscle symptoms after starting statins or fibrates. Rhabdomyolysis may occur with mild symptoms and signs and may occur later, even after discontinuation of the drug.

## Background

Dyslipidemia is an important risk factor for cardiovascular (CV) disease [1, 2]. Statins are the mainstay in lipid-lowering therapy. The combination of a statin and a fibrate, particularly in mixed dyslipidemia, offers greater lipid-modifying efficacy. Statins and fibrates are known to cause rhabdomyolysis [3–8]. Combination therapy of a statin and a fibrate increases the risk mainly due to pharmacodynamic interactions [9–12]. Advanced age, diabetes, renal impairment, hypothyroidism, and type and higher doses of statins are recognized as important risk factors of rhabdomyolysis [13–26]. Rhabdomyolysis may present with subtle symptoms and signs and may be delayed at onset even after stopping of the culprit agent. Aggressive fluid resuscitation and urine alkalinization are important management strategies. Myoglobin is not dialyzable owing to its larger size. However, a small subset of patients with rhabdomyolysis might need dialysis for renal indications such as hyperkalemia, acidosis, or fluid overload not responding to medical therapy [27–32]. We report a case of rhabdomyolysis leading to acute kidney injury mandating urgent hemodialysis following treatment with atorvastatin-gemfibrozil combination.

## Case presentation

We report the case of a 62-year-old Sri Lankan Sinhalese man who presented to our Teaching Hospital, Peradeniya with a history of generalized malaise and anorexia of 1 week’s duration.

He had had poorly controlled type 2 diabetes for 12 years treated with orally administered hypoglycemic agents, which was complicated by diabetic nephropathy with a baseline serum creatinine of 220 micromol/L which was done 3 months back with urine analysis showing ++ protein. He was on thyroxine 100 mcg for hypothyroidism. He was a hypertensive for 10 years on losartan potassium. He was dyslipidemic and was on orally administered atorvastatin 40 mg daily. Serum lipids done 1 month back revealed high serum cholesterol with elevated triglycerides (TG) of 350 mg/dL. Gemfibrozil 600 mg twice daily was added on top of the statin by his general practitioner. Soon after starting gemfibrozil, our patient developed myalgia and he himself stopped taking gemfibrozil after 5 days. He then noticed gradual resolution of symptoms.

He developed generalized malaise and anorexia 3 weeks after the initial event. He noticed a reduction in urine output with dark urine for 2 days prior to admission. He noticed a reduction in exercise capacity with shortness of breath at rest.

On examination his temperature was 36.7 °C (98 °F). He was oriented in time, place, and person. He had mild pallor and bilateral ankle swelling. His pulse rate was 96 beats/minute with a blood pressure of 140/80 mmHg; his precordium examination was normal. He was dyspneic at rest with a respiratory rate of 24 cycles per minute and he had bilateral fine crackles up to mid zone. An abdominal examination was unremarkable. He had mild tenderness of his thigh muscles. He had 4/5 of normal strength against resistance in proximal muscles and his distal muscles had strength close to normal. Deep tendon reflexes were present but diminished.

Laboratory investigations revealed a serum creatinine of 1232 micromol/L with a blood urea of 27.7 mmol/L. His serum potassium was 8 mmol/L with electrocardiographic evidence of hyperkalemia. His serum alanine transaminase (ALT) was 413 U/L and aspartate transaminase (AST) was 229 U/L. Hemoglobin was 9.4 g/dL with a mean corpuscular volume (MCV) of 85 fL. His white cell count was 6.6 × 10^6^/microL and platelet count of 87 × 10^3^/microL. Corrected serum calcium value was 1.63 mmol/L for an albumin value of 31 g/L. His serum phosphate was 4.7 mg/dL (2.5–4.5 mg/dL). His lactate dehydrogenase level was 240 U/L (88–230 U/L) and uric acid was 12.4 mg/dL (2.4–7.4 mg/dL). His C-reactive protein was 68.5 mg/L. Arterial blood gas revealed severe metabolic acidosis with a pH of 7.12: bicarbonate 8.8 mmol/L, partial pressure of carbon dioxide (PCO_2_) 27 mmHg, and base excess of − 20.5 mmol/L. An ultrasound scan of his kidney-ureter-bladder revealed normal size kidneys with slightly altered corticomedullary demarcation and no obstruction to outflow tract. His creatine kinase (CK) level was 49,146 U/L and urine myoglobin was positive. A diagnosis of rhabdomyolysis leading to acute-onset chronic renal failure was made. His random blood sugar was 145 mg/dL and urine ketone bodies were negative. He was clinically euthyroid while on thyroxine 100 mcg/day and his thyroid-stimulating hormone (TSH) was 6.5 u/L (normal range 0.4–4 u/L). Other causes of rhabdomyolysis, such as hyperthermia, prolonged seizures, trauma, physical muscle damage or stress, dehydration, burns, or alcohol abuse were not evident in our patient. He did not have fever preceding the event. His clinical course and laboratory data suggest that the combination of atorvastatin and gemfibrozil is the most probable cause of rhabdomyolysis.

He was catheterized. He was given calcium gluconate and insulin dextrose to manage the hyperkalemia. Acidosis was corrected with an 8.4% sodium bicarbonate infusion. He was started on intravenously administered furosemide. Considering his high serum creatinine, severe metabolic acidosis, and hyperkalemia, he was offered urgent hemodialysis via a vascular catheter. His daily renal functions and CK were monitored. He was offered four consecutive hemodialysis sessions every other day. Gradual improvement in his urine output was noted. His CK levels normalized over the days. He was discharged on day 8 of admission with a serum creatinine of 280 micromol/L and normal CK level. On a follow-up visit 1 week later, his serum creatinine was 256 micromol/L with normal CK level.

## Discussion

CV diseases are a major cause of morbidity and mortality in the world. According to the World Health Organization (WHO), the prevalence of CV diseases will double by 2020 and will rank higher than HIV/AIDS infection [1]. Dyslipidemia is recognized as an important risk factor for CV disease [2]. Statin therapy is recommended as the primary pharmacologic agent to achieve target low-density lipoprotein cholesterol (LDL-C) goals on the basis of morbidity and mortality outcome trials. Fibrates may improve atherosclerotic CV disease (ASCVD) outcomes in primary and secondary prevention when TG concentrations are ≥ 200 mg/dL and high-density lipoprotein cholesterol (HDL-C) concentrations are < 40 mg/dL [33].

Rhabdomyolysis is a clinical syndrome in which muscle necrosis leads to release of intramuscular contents into systemic circulation. It has a diverse clinical spectrum ranging from asymptomatic elevation of serum muscle enzymes to life-threatening cases associated with extremely high enzyme levels, electrolyte imbalances, and acute renal failure [3]. Patients may complain of myalgia, muscle weakness, or “tea color” urine. Diagnosis of rhabdomyolysis is often confirmed by elevated serum CK and myoglobinuria [4]. The incidence of rhabdomyolysis from all causes is 1.6 per 100,000 person-years [5].

Statins are 3-hydroxy-3-methylglutaryl coenzyme A reductase inhibitors. Statins are associated with a spectrum of skeletal muscle complaints ranging from asymptomatic elevation of serum CK, myalgia, and muscle cramps and weakness to severe myositis leading to rhabdomyolysis. Persistent myalgia and CK elevations are noted in some patients even after statin withdrawal [6]. The US Food and Drug Administration Adverse Event Reporting System database reports rates of statin-induced rhabdomyolysis of 0.3–13.5 cases per 1,000,000 statin prescriptions [7]. The mechanism of the myotoxic effects of statins is unclear. Statin-induced depletion of isoprenoids and inactivation of small GTPases, especially Rab, are postulated as important mechanisms in statin-induced myotoxicity [8].

The mechanism of rhabdomyolysis associated with fibrate therapy remains unclear. Although a few cases reported rhabdomyolysis in fibrate monotherapy, most of the cases are associated with statin and fibrate, particularly the gemfibrozil combination. Maiguma *et al.* described fibrate-medicated activation of the nuclear receptor peroxisome proliferator-activated receptor-A leading to cell-specific injury to human embryonal rhabdomyosarcoma cells *in vitro* to support a mechanism of fibrate-induced rhabdomyolysis [9]. Gemfibrozil and other fibrates are metabolized by CYP3A4 [10]; however, fibrates do not cause significant inhibition of the enzyme [11]. Pierce *et al.* reported the plasma levels of active metabolites of lovastatin were not usually elevated in 12 patients with severe myopathy who were receiving combined treatment with gemfibrozil supporting this fact [12]. The interaction between fibrates and statins is thought to be pharmacodynamic rather than pharmacokinetic. Therefore the rhabdomyolysis effect may be increased due to summation and synergism of myotoxicity of both drugs.

Many risk factors have being described in relation to statin-induced rhabdomyolysis. The risk of statin-induced muscle toxicity is more likely with higher doses [13]. In the Study of the Effectiveness of Additional Reductions in Cholesterol and Homocysteine (SEARCH) trial, in which 12,064 patients with a history of myocardial infarction were treated with simvastatin 20 mg or 80 mg daily (mean follow-up 6.7 years), the incidence of myopathy and rhabdomyolysis in patients on 80 mg/day were significantly high [14]. Advanced age and female sex were identified as predisposing factors potentially increasing the risk of statin-induced myopathy [15]. Six statins are currently prescribed: rosuvastatin, atorvastatin, simvastatin, pravastatin, fluvastatin, and lovastatin. Cerivastatin was taken off the market by its manufacturer in 2001, as many cases reported cerivastatin-induced rhabdomyolysis [16]. The risk of myopathy does not appear to be significantly different among all the other statins currently available; however, simvastatin at maximal dose of 80 mg was reported to cause more myotoxicity compared to other statins [17]. The risk for serious muscle toxicity appears to be increased in patients with diabetes, particularly in those with background renal impairment [18, 19]. Hypothyroidism increases the risk of statin and/or fibrate-induced rhabdomyolysis [20–22].

Drugs which interfere with statin metabolism or transport leading to increased systemic exposure to the drug increase the risk of myopathy. Nearly 60% of statin-related rhabdomyolysis is related to drug interactions [23]. Many statin–drug interactions involve the CYP3A4/5 (simvastatin, lovastatin, and atorvastatin) or CYP2C8/9 (rosuvastatin) drug metabolizing systems [24]. Some were modulated by the efflux transporter P-glycoprotein [25]. Some drugs interfere with the transporters responsible for hepatic uptake and excretion of statins may also affect the pharmacokinetics of statins and the risk of myopathy [26]. As discussed before, some are pharmacodynamic interactions with summative and synergistic properties of myotoxicity.

Few case reports in the literature describe the occurrence of rhabdomyolysis in patients who were stable with a single lipid-lowering agent, either a statin or a fibrate, and addition of a second agent had initiated myositis and subsequent rhabdomyolysis. Lau *et al.* described a case of rhabdomyolysis in a patient on long-term fibrate following addition of cerivastatin [27]. Another report by Jozić *et al*. described rhabdomyolysis in a patient in whom rosuvastatin was replaced with pravastatin and gemfibrozil [28]. Similar to our case, the patient was on long-term high-dose statin and occurrence of rhabdomyolysis followed the addition of a second lipid-lowering agent.

Our patient had many risk factors including advanced age, poorly controlled type 2 diabetes mellitus, background renal impairment, and hypothyroidism. He was on a large dose of statin that was subsequently combined with gemfibrozil resulting in massive rhabdomyolysis. He experienced muscle symptoms soon after adding gemfibrozil and the drug was discontinued. Although his symptoms settled soon after discontinuation, he presented to a health care institution after 4 weeks with uremic symptoms and anuria with a massive elevation of CK. This is the first case reporting rhabdomyolysis with renal impairment presenting 4 weeks after discontinuing fibrate after muscle symptoms. Gemfibrozil is a drug which is extensively metabolized by the liver and excreted mainly by the kidneys with an elimination half-life of 1.5 hours [29]. Underlying chronic renal impairment might have prolonged the elimination half-life. We believe that the summative and synergistic properties of statin and fibrate initiated the muscle damage. Initial myotoxicity would have propagated insidiously over the period of 4 weeks culminating in renal failure with anuria. The damaged muscle would have been more vulnerable to high-dose statin, which our patient continued to take until admission. Gradually worsening renal impairment due to the ongoing rhabdomyolysis could be a vicious cycle leading to more renal damage from the retaining myotoxins. Interestingly, he had minimum muscle symptoms and mild tenderness of thigh muscles despite having a CK of nearly 50,000 U/L.

The main step in managing rhabdomyolysis-induced acute kidney injury remains the early, aggressive repletion of fluids, but unfortunately it was not an option for our patient who was already in anuric renal failure [30]. Beall *et al*. proposed administering sodium bicarbonate, which results in an alkaline urine [34]. Alkalinization of urine is known to be advantageous in rhabdomyolysis [31]. First, it is known that precipitation of the Tamm–Horsfall protein–myoglobin complex is increased in acidic urine [32, 33]. Second, alkalinization inhibits reduction–oxidation (redox) cycling of myoglobin and lipid peroxidation in rhabdomyolysis, thus ameliorating tubule injury [35]. Third, it has been shown that metmyoglobin induces vasoconstriction only in an acidic medium in the isolated perfused kidney [36]. Our patient was administered 8.4% sodium bicarbonate to correct severe metabolic acidosis. However, since our patient was anuric, whether the above mentioned mechanisms operated is doubtful.

Conventional hemodialysis does not remove myoglobin effectively owing to the size of the protein. When acute kidney injury is severe enough with refractory hyperkalemia, acidosis, or volume overload, renal replacement therapy (RRT) is indicated, principally with intermittent hemodialysis [37]. Therefore the need of RRT should be mandated by renal indications. Our patient had a serum creatinine of more than 1000 micromol/L with severe hyperkalemia and severe metabolic acidosis with features of volume overload and anuria. He needed immediate hemodialysis to correct metabolic derangements. Gradually, he became normouric and the intake was titrated accordingly. Following four cycles of hemodialysis, the urine output of our patient improved, acidosis was corrected, and potassium was normalized. His serum creatinine level declined to 800 mmol/L. Dialysis was withheld and a gradual decline in CK levels and serum creatinine was noticed over the days.

## Conclusions

Rhabdomyolysis is a rare but serious side effect of statin monotherapy and more common in combination therapy of statin and fibrate. Patients with multiple risk factors such as diabetes, renal impairment, hypothyroidism, and polypharmacy are at increased risk of rhabdomyolysis. Rhabdomyolysis may occur with minimum clinical symptoms and signs, even many weeks after discontinuation of therapy. Treating physicians should be more cautious in prescribing statins or combination therapy of statin and fibrates in those with risk factors and should discuss signs and symptoms of muscle toxicity with patients in order to prevent rhabdomyolysis.
